# Mechanisms of Adiponectin Action

**DOI:** 10.3390/ijms20122894

**Published:** 2019-06-13

**Authors:** Tania Fiaschi

**Affiliations:** Dipartimento di Scienze Biomediche, Sperimentali e Cliniche “M. Serio”, Università degli Studi di Firenze, Viale Morgagni 50, 50134 Firenze, Italy; tania.fiaschi@unifi.it; Tel.: +39-055-275-1233

Adiponectin, the most abundant secreted adipokine, has received great attention from the scientific community since its discovery [1]. The huge number of studies is justified by the insulin-sensitizing role and the beneficial effects of adiponectin in diabetic conditions [2,3]. Over the years, a large body of evidence has supported a pleiotropic role of the hormone in different tissues in which it influences varied physiological aspects both in healthy and in diseased conditions. 

This Special Issue, entitled “Mechanisms of Adiponectin Action”, shows the pleiotropic role of adiponectin by presenting three research articles and seven reviews focused on recent findings about adiponectin in different target tissues. The Special Issue contains two reviews about adiponectin in skeletal muscle, a classical adiponectin target tissue, in which the hormone affects both metabolic [4] and regenerative properties [5]. Krause et al. report a close relationship between physical exercise and expression and circulating levels of adiponectin in both healthy and diseased population. Indeed, a higher adiponectin level is associated with greater physical activity, while some conditions, such as inactive obese, pre-diabetic, and diabetic patients, are characterized by decreased adiponectin levels. The restoration of proper adiponectin levels can be achieved with physical exercise, and this leads to increased insulin sensitivity [6]. Gamberi et al. discusses the current knowledge about adiponectin in myopathies (both non-inherited/acquired and inherited myopathies). The paper reports that some myopathies (as Duchenne muscular dystrophy and collagen VI-related myopathies) are characterized by a decreased circulating adiponectin level and that hormone replenishment induces beneficial effects in the diseased muscles [7]. Studies about the involvement of adiponectin in cancer have been growing in the last years. Parida et al. report how obesity and adiposity are closely related to cancer progression in several types of tumors (such as liver, pancreatic, prostate, and colorectal cancers). Obesity acts by dysregulating adipokine production, leading to the upregulation of oncogenic adipokines, such as leptin, and the downregulation of adiponectin, which plays a protective role in obesity-associated cancers [8]. About the relationship between obesity and cancer, Gelsomino et al. describe the role of adiponectin in the onset of obesity-associated female cancers (such as cervical, ovarian, endometrial, and breast cancers), reporting that adiponectin exerts anti-proliferative actions in some female cancers [9]. Barbe et al. report an overview of the expression levels and signaling pathways of adiponectin in male and female reproductive tract, from gametogenesis to embryo implantation and embryonal development. In addition, the authors describe some diseases associated with infertility, characterized by altered adiponectin levels (such as polycystic ovary syndrome, ovarian and endometrial cancers, endometriosis, gestational diseases, preeclampsia, and foetal growth restriction) [10]. Smolinska et al. report a research article describing a comparative transcriptomic study performed in control and adiponectin-treated endometrial tissues isolated from 15- to 16-day-pregnant pigs. The findings evidence that adiponectin affects processes important for reproductive success, such as cell proliferation, cell adhesion, and synthesis of steroids, prostaglandins, and cytokines [11]. The anti-atherogenic role of adiponectin has been widely recognized. Yanai et al. illustrate the mechanisms underlying the anti-atherogenic role of the hormone, describing methods (such as weight loss, exercise, administration of nutritional factors and anti-diabetic drugs) leading to the rise of circulating adiponectin, which have been proven to have protective effects against atherosclerotic progression [12]. A role of adiponectin in the mitigation of renal injury due to diabetes has been proposed. Kim et al. highlight recent advances about adiponectin and kidney diseases, describing adiponectin signaling pathways in healthy and disease conditions. In particular, the review discusses the possible strategies for upregulating adiponectin and adiponectin receptors and the possible use of the receptor agonist AdipoRon in the amelioration of overt diabetic kidney disease [13]. Furthermore, this Special Issue contains two research articles regarding adiponectin involvement in human follicular dermal papilla cells and the interaction of adiponectin with nerve growth factor β (NGFβ) and secreted protein acidic and rich in cysteine (SPARC). Park et al. report that kojyl cinnamate ester derivatives and Seletinoid G promote adiponectin secretion by human follicular dermal papilla cells. In addition, cell medium containing secreted adiponectin induces the expression of hair growth-related factors, thus suggesting an involvement of the hormone in the promotion of hair growth in humans [14]. Finally, Okura et al. investigate the interaction between adiponectin and NGFβ and SPARC. Surface plasmon resonance analysis demonstrated a physical interaction between adiponectin and NGFβ, and this interaction was confirmed in neuronal cultured PC12 cells [15]. 

Collectively, the papers reported in this Special Issue reinforce the idea that adiponectin plays an important role at the systemic level and that hypoadiponectinemia is associated with many diseases. The exogenous administration of adiponectin has often beneficial effects in diseased tissues, suggesting that the planning of new drugs able to activate adiponectin signaling could be a new tool for the amelioration of several pathologies.
